# An exploratory pilot study of artificial intelligence-based instructional program for developing productive thinking skills among female King Faisal University students

**DOI:** 10.3389/fpsyg.2026.1812540

**Published:** 2026-08-24

**Authors:** Hissah Fahad AlMuhaysh

**Affiliations:** Department of Curricula and Teaching Methods, College of Education, King Faisal University, Al-Ahsa, Saudi Arabia

**Keywords:** artificial intelligence, educational technology, instructional program, productive thinking, university students

## Abstract

**Introduction:**

This study aimed to investigate the effectiveness of a teaching program based on artificial intelligence in developing productive thinking skills among female university students.

**Methods:**

The study adopted a quasi-experimental design with a single experimental group. The participants were nine female students enrolled in the Educational Technology course at the College of Sharia, King Faisal University, in the Al-Ahsa Governorate, during the first semester of the 2024–2025 academic year. The sample was purposefully selected from the study population. The instrument used was a Productive Thinking Skills Questionnaire consisting of 20 items distributed across seven core skills: originality, fluency, flexibility, inference, interpretation, expansion, and imagination. To address psychometric robustness, face validity was established via a panel of five expert judges, and construct validity was substantiated by assessing item-total correlations alongside the internal consistency coefficient. A five-point Likert scale was used to score the responses, ranging from “Needs Improvement” to “Excellent.” The reliability of the scale was confirmed with a Cronbach’s alpha coefficient of 0.84. The study applied a teaching program based on artificial intelligence, and data were analyzed using the R statistical software through descriptive and inferential statistics, including means, standard deviations, effect sizes, Cronbach’s alpha, and the Wilcoxon signed-rank test due to the small sample size. Confidence intervals (95% CI) for median differences were computed using exact distribution methods.

**Results:**

The results revealed statistically significant improvements between pre- and post-test scores, suggesting the potential effectiveness of the program within this exploratory pilot context.

**Discussion:**

Given the small sample size (*n = 9*), this study is framed as a pilot design; the findings are associative and exploratory, intended to generate preliminary evidence rather than definitive causal inferences. The critical limitations of the single-group design—such as vulnerability to maturation, testing effects, and task familiarity—are extensively evaluated. The study recommends incorporating artificial intelligence-based teaching strategies in university education to enhance students’ productive thinking skills.

## 1 Introduction

Rapid advances in artificial intelligence (AI) are reshaping higher education worldwide, compelling universities to reconsider how learning environments can better support higher-order cognitive skills required in the twenty-first century (Kaufman and Beghetto, 2013). Educational institutions are increasingly expected not only to integrate digital technologies but also to leverage them strategically to foster creativity, critical thinking, and problem-solving capacities essential for academic, professional, and societal participation (U.S. Department of Education and Office of Educational Technology, 2023). In this context, AI has emerged as a particularly influential educational innovation, offering unprecedented opportunities for personalized instruction, adaptive feedback, and learner-centered pedagogical design (Ng et al., 2023).

Recent years have witnessed a rapid expansion of AI-supported educational applications, especially following the emergence of advanced generative AI tools capable of producing text, feedback, and problem-solving suggestions in real time (Beatty et al., 2018). These developments have intensified scholarly interest in AI’s potential to enhance learning outcomes and cognitive engagement across disciplines (Garzón et al., 2025). At the same time, scholars have emphasized the growing importance of AI literacy as a foundational competence, enabling students to critically interact with intelligent systems rather than passively consume their outputs (Chiu et al., 2024). This emerging competency, broadly conceptualized as AI literacy, functions as an essential cognitive scaffold that allows students to interact dynamically with automated systems, interrogate algorithmic outputs, and co-construct more sophisticated conceptual products rather than accepting AI outputs uncritically. Within higher education, AI is increasingly viewed not merely as a technological aid but as a pedagogical framework capable of transforming learning processes and supporting the development of complex thinking skills (Ritchhart and Perkins, 2005).

Among these skills, productive thinking occupies a central position. Productive thinking represents an integrative cognitive process that combines creative and critical thinking to generate effective, novel, and contextually appropriate solutions (Benedek et al., 2014; Hurson, 2007). It encompasses multiple interrelated dimensions, including fluency, flexibility, originality, inference, interpretation, expansion, and imagination, which collectively enable learners to analyze problems, generate alternatives, and translate ideas into actionable outcomes. Prior research has demonstrated that productive thinking is closely associated with academic success, adaptive decision-making, and innovation across educational and professional contexts (Basadur et al., 1982; Guilford, 1967).

In the realm of educational psychology, recent work emphasizes that interactive learning environments driven by generative tools can effectively trigger metacognitive monitoring and recursive thinking patterns (Song and Cai, 2024). When undergraduate students engage with generative feedback, they are prompted to confront a degree of cognitive disequilibrium, which can stimulate the reconfiguration of internal mental schemata and drive the multi-perspective exploration characteristic of productive thinking.

Theoretical and empirical studies suggest that productive thinking skills are not fixed traits but can be deliberately developed through structured instructional interventions. Guilford’s (1967) foundational work on divergent thinking identified fluency, flexibility, originality, and elaboration as core components of creative cognition, while later research emphasized the role of interpretation and inference in enabling deeper understanding and problem framing (Li et al., 2022). Contemporary studies further confirm that learning environments encouraging exploration, reflection, and real-world application are particularly effective in fostering productive thinking among university students (Aladwan, 2024; Lee and Lee, 2024; Song and Cai, 2024).

Within this evolving landscape, AI-supported learning environments have attracted increasing attention as potential catalysts for productive thinking development. Machine learning algorithms and generative systems can expand traditional brainstorming processes, offer alternative perspectives, and provide immediate feedback that supports iterative idea refinement (Jordan and Mitchell, 2015; Oxman, 2006). Moreover, AI-enabled platforms can adapt learning pathways to individual cognitive profiles, thereby enhancing engagement and supporting diverse thinking strategies (Ouyang and Jiao, 2021). These features position AI as a promising medium for nurturing the multidimensional processes underlying productive thinking.

Despite growing interest in AI-enhanced education, empirical research examining how AI-based instructional programs influence productive thinking in authentic university contexts remains limited, particularly within humanities-oriented disciplines and non-Western educational settings. Existing studies often focus on technical skills, computational thinking, or large-scale implementations, leaving a gap in understanding the feasibility and cognitive impact of AI-supported instruction in small, context-specific learning environments (Toplak et al., 2014). Furthermore, few studies explicitly examine the development of productive thinking as a multidimensional construct within AI-enhanced pedagogy, and even fewer address gender-specific or culturally situated learning contexts.

To address these gaps, the present study explores the implementation of an AI-based instructional program designed to develop productive thinking skills among female undergraduate students at King Faisal University. Situated within an educational technology course at the College of Sharia, the study adopts an exploratory pilot design to examine whether structured engagement with AI tools can support measurable improvements across the seven dimensions of productive thinking. By focusing on a real-world university setting and explicitly framing the investigation as a pilot study, this research aims to generate preliminary empirical evidence regarding the feasibility, potential effectiveness, and pedagogical implications of AI-supported instruction for productive thinking development.

In doing so, the study contributes to the growing interdisciplinary literature on AI in education by offering context-sensitive insights into how intelligent learning environments can support higher-order cognitive skills. Rather than advancing definitive causal claims, the findings aim to inform future large-scale and comparative research, refine instructional design principles, and support the evidence-based integration of AI technologies in higher education.

## 2 Literature review

The student’s role in contemporary education is not limited to recalling stored knowledge but extends to generating new meanings and innovative applications through productive thinking. This pattern of thinking, which encompasses the ability to create novel ideas, solve complex problems creatively, and synthesize information in creative ways, has been recognized as fundamental to cognitive development since the pioneering work of researchers in the mid-20th century. Relevant studies in the literature have confirmed that productive thinking is closely linked to the ability to adapt to developments and make effective decisions in complex situations (Basadur et al., 1982; Guilford, 1967). Guilford’s seminal work on divergent thinking established the theoretical foundation for understanding creativity as a measurable cognitive ability, identifying key dimensions such as fluency, flexibility, originality, and elaboration that collectively contribute to productive thought processes. Building on this foundation, contemporary research has demonstrated that productive thinking skills can be deliberately developed through structured educational interventions and training programs, enabling learners to enhance their capacity for innovation and creative problem-solving across diverse contexts (Aladwan, 2024; Lee and Lee, 2024). Recent studies further emphasize that fostering productive thinking in educational settings requires creating learning environments that encourage exploration, experimentation, and the application of knowledge to real-world challenges, thereby preparing students to navigate the complexities of the twenty-first century (Song and Cai, 2024).

Recent research has identified several dimensions of productive thinking. For example, fluency refers to a student’s ability to produce a range of ideas or alternatives in a complex or challenging situation. As more ideas are generated, the greater the chances of reaching effective and innovative solutions become (Runco and Jaeger, 2012).

Flexibility is the ability to navigate between multiple thought patterns and strategies when facing a single problem, giving due consideration to atypical and unconventional solutions as viable alternatives (Chen et al., 2014). The importance of flexibility becomes evident when a problem is surrounded by a diversity of variables, as the student learns to adjust her thinking path in response to developments quickly (Tong et al., 2023).

The dimension of originality reflects the student’s ability to generate unfamiliar and innovative ideas, distinct from common and obvious solutions. Originality is not merely a deviation from the familiar but an added value characterized by innovation and novelty. Kim (2006) noted that the Torrance Test, which measures creative thinking, assesses originality by comparing the uncommon quality of answers to the standard responses of the statistical community, considering originality to be an accurate indicator of the level of innovation among individuals (Kim, 2006).

The dimension of interpretation involves understanding the implications of information and data, clarifying their meanings in different contexts, and the ability to extract meaning from texts and situations, linking them to broader conceptual frameworks. Interpretation enables students to comprehend the hidden dimensions of the problem and prepares them to formulate new research questions or hypotheses (Li et al., 2022).

Inference concerns the ability to draw logical conclusions based on given data or hypotheses, and to recognize their interconnections to generate new insights and deductions. Inference serves as a bridge between explicit and implicit knowledge. Within the framework of critical and creative thinking, inference enables students to build solution strategies based on rational foundations. Therefore, it is a fundamental pillar in the advanced stages of productive thinking. Studies within the framework of critical thinking confirm that interpretation, accompanied by inference, constitutes the essential element for understanding a problem in depth before generating solutions (Li et al., 2022).

Meanwhile, the dimension of expansion refers to an individual’s ability to develop a specific idea by adding more details and depth, essentially expanding on the idea or solution and explaining how it can be applied. Within the framework of productive thinking, expansion adds substance and realism to creative ideas, helping to clarify the feasibility of the solution and its effectiveness. In this way, the skill of expansion enables students to find comprehensive and applicable solutions, develop them, and thereby enhance the quality and seriousness of their creative ideas in educational situations (Guilford, 1967).

Finally, the dimension of imagination refers to the mental ability to form new images or concepts that are not directly related to present reality, which is an essential element in the creative thinking process. The Programme for International Student Assessment (PISA) (Organisation for Economic Co-operation and Development [OECD], 2022) has confirmed that the attributes of imagination and intellectual openness are positively associated with the efficacy of creative thinking; data from the OECD report that students who feel they are “imaginative” and curious tend to achieve higher performance in creative thinking skills. In other words, imagination works to broaden the boundaries of students’ thinking and cultivate a fertile imagination that contributes to producing innovative and creative solutions, thereby giving it great educational value in refining productive thinking skills.

Thus, productive thinking encompasses integrated dimensions that support intelligent learning environments informed by AI. Algorithms work to monitor fluency, provide activities that enhance flexibility, evaluate the originality of ideas, and then motivate students to make inferences and interpretations through interactive situations that offer immediate feedback. This systematic application of the seven dimensions is designed to foster the development of productive thinking skills among female students at King Faisal University. In the context of accelerated growth, AI has emerged as a stimulating factor that reshapes the features of university learning environments and gives productive thinking new dimensions. Research has shown that machine learning-based algorithms can scan the vast field of ideas and provide initial suggestions that enrich traditional brainstorming sessions, expanding the range of creative possibilities for students (Jordan and Mitchell, 2015). Additionally, AI-supported design environments, such as tools based on generative neural networks, enable learners to transform their mental visions into interactive prototypes more quickly, thereby shortening development cycles and enhancing the interaction between the mind and design material (Oxman, 2006).

Sun et al. (2021) view AI as combining mental capabilities manifested in displaying intelligent behaviors and making decisions that simulate human performance. AI is “a field that aims to design and develop systems capable of performing tasks of a cognitive nature similar to humans, such as learning, reasoning, and adapting to new situations” (Russell and Norvig, 2020, 25). In the context of higher education, this definition assumes a practical dimension that can be broken down into three principal axes: adaptive customization of educational pathways, intelligent educational guidance, and early detection of performance patterns (Nijstad et al., 2010).

Regarding the contribution of AI to the development of productive thinking, the literature reveals that algorithms based on generative neural networks provide initial suggestions that support the brainstorming stages, thereby expanding the range of creative possibilities for students (Jordan and Mitchell, 2015). Computational design tools also help researchers transform mental conceptions into three-dimensional models, reducing the time required for design iteration and enhancing the interaction between idea and implementation (Oxman, 2006). In addition, AI-supported collaboration platforms coordinate virtual teams by analyzing members’ experiences and the volume of their previous contributions, guiding discussion toward innovative solutions and higher effectiveness in managing research projects (Woolf, 2010).

The results of studies by Trelease (2014), Van Nuland and Rogers (2015), Yoo and Huang (2016) suggest that computer technologies play a crucial role in enhancing the educational process and refining the function of education. Computer programs are widely used today to achieve desired educational outcomes in various academic contexts, contributing to individuals’ awareness of new scientific methods for accessing required information more efficiently, thanks to the incorporation of computational methods and techniques into educational curricula.

The results of studies by Celik et al. (2022), Valtonen et al. (2021) indicate that teachers’ use of AI applications contributes to developing their teaching skills, helping them to understand students’ needs better, select appropriate educational content and activities, and evaluate and monitor students’ progress while providing immediate feedback. This enhances the development of 21st-century skills among both students and teachers, demonstrating the effectiveness of employing AI applications in achieving the objectives of teaching and learning processes.

The United Nations Educational, Scientific, and Cultural Organization (UNESCO) emphasizes the necessity of applying AI tools in education to promote sustainable development through effective collaboration between learners and computers in the learning process, as well as in life and work. Recommendations from the 2019 International Conference on Artificial Intelligence and Education, held in Beijing, also emphasize the potential of using AI in education across several key areas, including the development of teacher and learner skills, as well as social skills such as values, life skills, and lifelong learning skills (UNESCO, 2019).

AI is theoretically based on several educational theories; it can be classified into three patterns in education according to these theories. The first is directed AI (behaviorism), which aligns with the principles of behavioral learning theory by focusing on the connection between stimuli and responses and using punishment and reinforcement to shape behavior. Here, AI acts as a teacher, providing learners with information, instructions, and feedback, measuring their performance, and determining their level, while learners are passive recipients of knowledge-oriented learning. The second pattern is supported AI (cognitive and social constructivist), which aligns with the principles of mental and social constructivist learning theory, focusing on building knowledge through interaction with the environment and others, and utilizing strategies such as exploration, discovery, and collaborative problem-solving. In this pattern, AI acts as an assistant that supports and stimulates learning, providing learners with resources, tools, and guidance to enhance their learning experience. It helps them organize, refine, and evaluate their knowledge, with learners being active collaborators in a collaboration-oriented learning environment. The third pattern is enabling AI (connectivism, a complex adaptive system), which aligns with the principles of adaptive learning theory. This approach focuses on allowing the learners to learn independently, innovatively, and responsibly by utilizing strategies such as project-based learning, goal-based learning, and choice-based learning. Here, AI serves as an enabler, providing a flexible, personalized, and diverse learning environment for learners, allowing them to control their path, pace, and learning method (Ouyang and Jiao, 2021, 3–5).

Li et al. (2017, 65) affirm that AI is “a scientific and technical current that includes methods, theories, and techniques aimed at creating machines capable of simulating human intelligence,” which views these three patterns as integrated tools for building flexible, interactive university learning environments responsive to the needs of female King Faisal University students.

Very recent evidence continues to refine this picture, indicating that generative AI’s contribution to higher-order thinking is conditional rather than automatic. In a mixed-methods analysis of university students’ ChatGPT interactions, Nasr et al. (2025) found that unguided use tended to remain confined to the earlier, exploratory stages of cognitive engagement, whereas structured, guided use allowed students to progress through evaluation and application, reframing the tool as a genuine cognitive partner rather than an answer machine. This finding reinforces the rationale for the present study’s emphasis on instructor-guided, scaffolded AI activities rather than unstructured AI access, and underscores the importance of examining how AI is used, not merely whether it is used, when interpreting gains in productive thinking.

From this perspective, it is clear that AI can function not only as an assistive technology but also as a supportive instructional framework that influences learning and thinking processes in specific educational contexts. AI is not limited to serving as an aid in university education but occupies an advanced research position that enables students to expand their perceptions and activate their productive capacities in an environment enhanced by intelligent technologies. The significance of this research lies in its potential to bridge the gap between theoretical understanding and practical application by providing a scientifically validated model that can be implemented and further refined in university settings. By focusing on the educational technology course in the Department of Religious Fundamentals at the College of Sharia during the first semester of the 2024–2025 academic year, the study offers a contextualized examination of how AI-supported learning environments can enable students to engage with complex problem situations, receive algorithm-enhanced personalized feedback, and follow individualized learning paths adapted to their specific cognitive patterns. This approach positions AI not as a peripheral educational tool but as a central framework for transforming pedagogical practices and fostering higher-order thinking skills that are essential for navigating the complexities of modern professional and academic contexts.

University education is an ideal environment for applying this study, especially in light of universities’ inclusion of digital transformation and adoption of AI technologies in their strategic plans. In this context, King Faisal University aims to develop modern educational programs that enhance the critical thinking and innovation skills of its female students. Based on this approach, the need to employ intelligent educational environments that rely on AI emerges, which can be an effective means of supporting the development of productive thinking among university students. This can be achieved by designing educational situations that require students to interact with real or virtual problem situations using algorithm-supported analysis tools to provide personalized feedback and individual learning paths that adapt to the thinking patterns of each student. Intelligent learning environments also offer opportunities for experimentation and exploration without fear of error, which represents an essential condition for unleashing the creative energy of female students. This underscores the importance of this study, which seeks to build an intelligent teaching program based on the latest concepts of AI, designed in an educational manner consistent with the characteristics of female students, and tested in terms of its effectiveness in enhancing and developing productive thinking skills, thereby contributing to providing a scientific model applicable and trainable in a university environment.

Collectively, constructivist, connectivist, and creativity-based perspectives provide a coherent explanatory framework for understanding how AI-supported learning environments foster productive thinking. Constructivism explains how learners actively build knowledge through interaction and reflection, while connectivism emphasizes learning through networks, digital tools, and information flows enabled by AI. Creativity models clarify how fluency, flexibility, originality, and imagination emerge through iterative idea generation and evaluation (Cropley, 2015). AI features such as adaptive feedback, generative reasoning, and personalization operate at the intersection of these theories, supporting students’ engagement across all seven dimensions of productive thinking. This integrated framework underpins the instructional design of the present study and explains the observed learning outcomes.

### 2.1 Study objectives

These aims directly align with the study’s dual objectives:

- Construct a pedagogically sound instructional program based on AI principles that specifically targets the development of productive thinking skills.
- Evaluate the effectiveness of the AI-based instructional program in enhancing productive thinking capabilities among female students at King Faisal University empirically.

## 3 Methodology

### 3.1 Main hypothesis

Within the context of rapid technological advancement and the accelerating global digital transformation, higher education institutions have increasingly adopted artificial intelligence–based applications as part of their instructional practices. In line with this direction, King Faisal University has sought to implement contemporary educational programs that enhance students’ cognitive skills, particularly those related to higher-order thinking and innovation. The present study was designed on the premise that the university learning environment provides a suitable context for examining the effectiveness of AI-based instructional interventions in developing productive thinking skills.

Productive thinking, which integrates both creative and critical thinking processes to achieve meaningful and practical outcomes, represents a key competency for university students navigating the complex demands of the 21st-century labor market. This construct encompasses multiple interrelated dimensions, including fluency, flexibility, originality, inference, interpretation, expansion, and imagination, which together form a comprehensive framework that can be systematically nurtured through AI-enhanced teaching strategies.

Accordingly, the central hypothesis of the study was formulated as follows:

There is no statistically significant difference at the significance level (α ≥ 0.05) between the mean scores of the experimental group students on the pre-test and post-test of the Productive Thinking Skills Scale.

### 3.2 Study design

This study employed a quasi-experimental design, specifically a single-group pre-test–post-test design, to investigate the effectiveness of an AI-based instructional program in enhancing productive thinking skills among female students at King Faisal University. Given the exploratory nature of implementing AI-based instruction in this specific educational context and the availability of participants, the study was conducted with nine female students as an initial investigation. This design allowed for a preliminary assessment of the program’s associative outcomes and feasibility.

The absence of a control group was a deliberate methodological decision aligned with the exploratory and pilot nature of the study. Institutional constraints, limited course enrollment, and ethical considerations related to equal access to innovative instructional practices prevented the formation of a comparison group. As such, the study was designed to generate preliminary evidence regarding feasibility and potential effectiveness rather than causal inference.

### 3.3 Participants

The study population consisted of all female students who enrolled in the educational technology course during the first semester of the 2024–2025 academic year at the College of Sharia, King Faisal University, in Al-Ahsa Governorate. The sample consisted of nine female students (*n* = 9) who were purposively selected from the study population to match the intervention’s objectives. Participation in the experimental group was limited to individuals who received the instructional program. Their ages ranged between 19 and 20 years, with a mean age of 19.4 years and a standard deviation of 0.5 years. All participants were female undergraduate students from the same academic program, reflecting a homogeneous sample in terms of educational background and cultural context. The internal and external validity limitations inherent to this small, single-group cohort are detailed comprehensively in the Limitations section.

The decision to utilize a sample of nine students was necessitated by institutional enrollment limits within the specialized Educational Technology course and the desire to maintain high instructional fidelity during this pilot phase. While this small n limits broad generalizability, the design serves as a foundational case study to explore the associative relationship between AI integration and cognitive development in a real-world higher education setting.

This sample size carries direct statistical consequences that should be made explicit. With *n* = 9, the design has adequate power to detect only large within-group effects and is underpowered for small or moderate ones; correspondingly, the 95% confidence intervals for the pre–post differences reported in Table 3 are wide, reflecting substantial sampling uncertainty around each point estimate. Readers should therefore treat the effect-size magnitudes as indicative of this exploratory pilot sample rather than as precise, generalizable parameters.

### 3.4 Procedure

**FIGURE 1:**
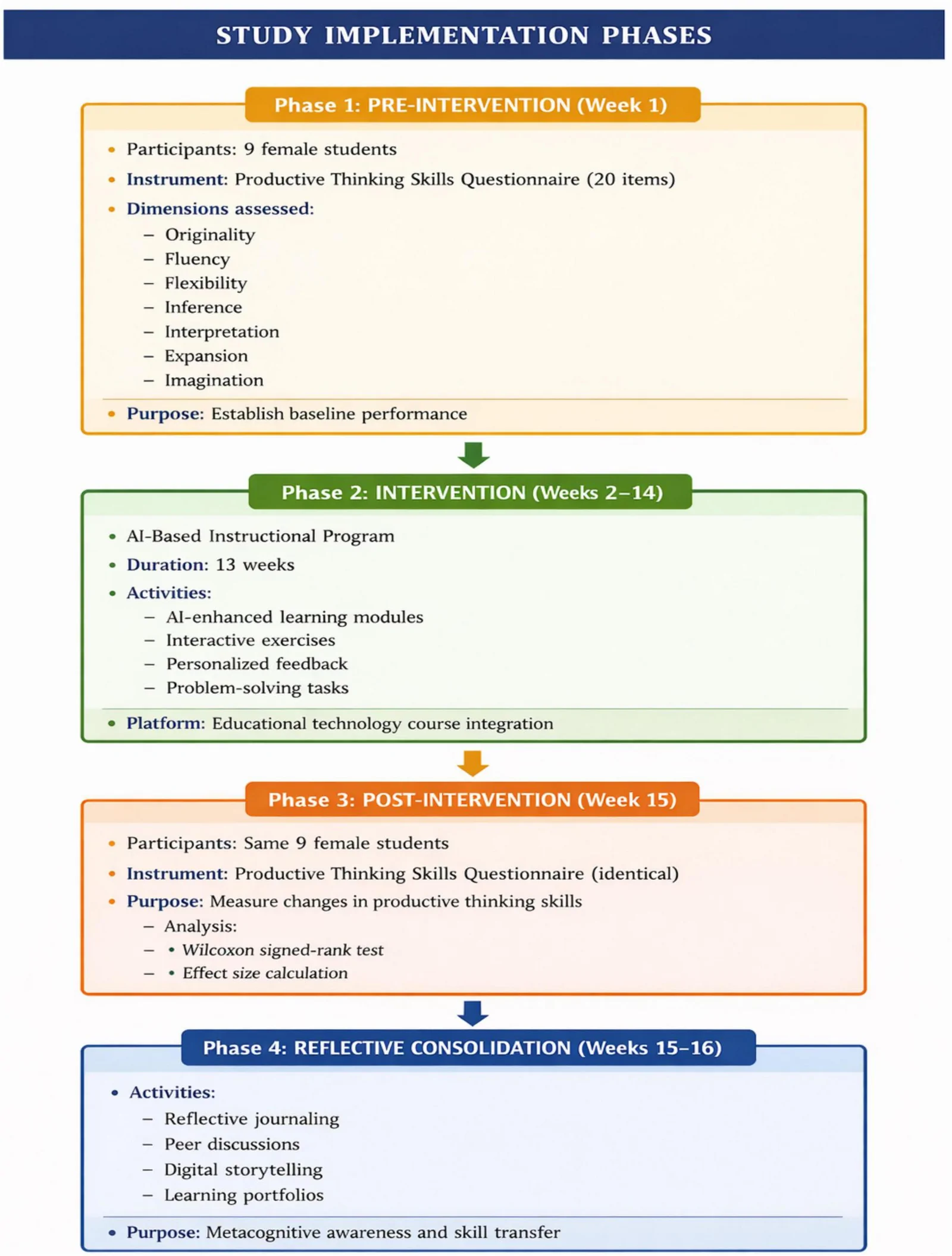
Study procedure and data collection timeline.

The study followed a systematic implementation process as illustrated in Figure 1. Before data collection, necessary approvals were obtained from the relevant institutional authorities, and informed consent was secured from all participants after explaining the study’s objectives, procedures, and ethical considerations.

As shown in Figure 1, the study was conducted over 16 weeks during the first semester of the 2024–2025 academic year. The pre-test was administered in Week 1 using the Productive Thinking Skills Questionnaire to establish baseline performance across seven dimensions. The AI-based instructional program was implemented over 13 weeks (Weeks 2–14), integrating AI-enhanced learning activities within the educational technology course. The post-test was administered in Week 15 using the same instrument to measure skill development. A reflective consolidation phase (Weeks 15–16) incorporated reflective journaling, peer discussions, and digital storytelling to foster metacognitive awareness and facilitate the transfer of skills to real-world contexts.

#### 3.4.1 Research instruments and data collection

All data were anonymized, coded, and processed for quantitative analysis in alignment with the ethical standards for educational research. Throughout the process, participants were provided with opportunities for feedback, reflection, and clarification to ensure the integrity and reliability of the intervention outcomes.

#### 3.4.2 Productive thinking skills

The present study employed a comprehensive psychometric instrument to assess participants’ productive thinking skills. The assessment tool, developed and validated by Salah (2024), was explicitly designed to measure the multidimensional construct of productive thinking through seven empirically derived dimensions: originality, fluency, flexibility, inference, interpretation, expansion, and imagination. The instrument comprises 20 items strategically distributed across these dimensions to ensure comprehensive coverage of the theoretical framework: originality (3), fluency (4), flexibility (2), inference (4), interpretation (2), expansion (3), and imagination (2) (Appendix).

All items presented respondents with five-point Likert-type options (1 = *needs development*, 2 = *developing*, 3 = *satisfactory*, 4 = *strong*, and 5 = *excellent*), enabling a nuanced evaluation of participants demonstrated productive thinking abilities. The cumulative score across all dimensions provides a composite index of productive thinking capability, with higher aggregate scores indicating more advanced productive thinking skills. The psychometric properties of the instrument were rigorously evaluated. Internal consistency reliability was assessed using Cronbach’s alpha coefficient. The overall reliability of the scale was high (α = 0.84), indicating strong internal consistency. Furthermore, all sub-dimensions demonstrated acceptable reliability levels, with alpha coefficients exceeding the recommended threshold of 0.70, confirming the adequacy of the instrument for research purposes. Table 1 presents the Cronbach’s alpha reliability coefficients for the total scale and each sub-dimension of the Productive Thinking Skills Questionnaire.

**TABLE 1:** Reliability coefficients of the sub-dimensions.

Dimension	Originality	Fluency	Flexibility	Inference	Interpretation	Expansion	Imagination	Total scale
Items	3	4	2	4	2	3	2	20
Cronbach’s alpha (α)	0.77	0.81	0.73	0.83	0.75	0.79	0.72	0.84

While the reliability evidence in the original submission focused solely on internal consistency (Total Scale α = 0.84), additional validation procedures were undertaken to strengthen the psychometric basis of the instrument. Face and content validity were established prior to the intervention by a panel of five expert judges in educational psychology and curriculum design, who evaluated each item for theoretical alignment, clarity, and cultural appropriateness; items falling below an 80% consensus threshold were revised accordingly. Construct validity was further examined through item-total correlation analysis conducted with a separate pilot group (*N* = 15) drawn from the same student population but not included in the main study. Pearson correlations between individual items and their respective sub-scale totals ranged from 0.58 to 0.79 (*p* < 0.01), indicating satisfactory item relevance and structural coherence. It should nonetheless be noted that this evidence, while stronger than internal consistency alone, still falls short of an independent, performance-based validation against an external criterion measure of productive thinking; consequently, post-test scores are best interpreted as self-perceived productive thinking rather than directly observed task performance. This distinction is revisited in the Limitations section.

The instrument was administered at two temporal points within the research design framework, once as a pre-intervention baseline measure and subsequently as a post-intervention assessment, to facilitate precise quantification of developmental changes in participants’ productive thinking abilities attributable to the AI-based instructional intervention program. This pre- and post-intervention approach allowed a robust comparative analysis of cognitive development outcomes across the experimental timeline.

#### 3.4.3 Exposure to the instructional program

The instructional intervention utilized a set of widely accessible generative and organizational artificial intelligence tools, including ChatGPT for idea generation, reasoning support, and feedback simulation, as well as Notion AI for structuring learning artifacts, facilitating reflective writing, and enabling collaborative documentation. These tools were selected due to their availability, ease of use, and relevance to higher education learning tasks. No custom-built AI systems were developed for this study; instead, commercially available platforms were integrated into instructional activities to reflect realistic classroom implementation.

#### 3.4.4 Instructional protocol and implementation fidelity

To address the level of procedural detail requested during review, this subsection specifies the AI interaction protocol underlying the six core sessions summarized above. Across the 13-week instructional window, students engaged with ChatGPT and Notion AI in guided, in-class blocks of approximately 45–60 min per session (a 10-min instructor modeling phase, 25–35 min of guided student prompting and AI interaction, and a 10–15 min debrief/reflection phase), supplemented by an expected minimum of two independent prompting cycles per week logged by students between sessions. The instructor reviewed AI-generated outputs with students before they were incorporated into deliverables, using a standing rubric addressing relevance, originality, and logical coherence rather than accepting AI outputs at face value. Table 2 summarizes the core activity and a representative (not exhaustive) prompt structure for each targeted dimension; the complete prompt bank is available from the corresponding author upon request.

**TABLE 2:** Illustrative AI interaction protocol by instructional session.

Session theme	Targeted skill	Core activity and AI interaction protocol	Exemplary prompt constraint
Session 2	Fluency	Students generated comprehensive lists of instructional challenges, using AI to brainstorm expansive solution fields within 15 min and tracking the quantity of viable alternatives.	“Act as an educational technology expert. Generate 15 distinct, non-overlapping pedagogical challenges associated with synchronous remote learning in higher education.”
Session 3	Originality	Students submitted common problem-solving ideas to AI, instructing the system to eliminate conventional responses and output statistically unusual or unconventional alternative frameworks.	“Analyze the attached list of standard student engagement techniques. Provide five highly unconventional, creative alternatives that leverage mixed-reality interfaces, ensuring no duplication of standard methods.”
Session 4	Flexibility	AI simulated shifting environmental or institutional constraints (e.g., sudden loss of funding, server crashes), requiring students to rapidly adjust their core project designs.	“Review my instructional design plan. Introduce three severe technical or administrative constraints into this scenario and prompt me to reformulate my strategy from a cognitive constructivist lens.”
Sessions 5 and 6	Interpretation and inference	Students entered raw, uninterpreted educational data into the AI system, generated multiple competing analytical hypotheses, and systematically examined the logical validity of each AI-generated interpretation.	“Given the following anonymized student survey metrics, draft three distinct interpretive hypotheses explaining the drop in motivation. Highlight any logical leaps or data insufficiencies in each hypothesis.”

#### 3.4.5 Illustrative example of AI-supported learning activity

To enhance methodological transparency, an example of a typical AI-supported interaction is provided. During the brainstorming session, students entered the following prompt into ChatGPT:

*“Generate five innovative instructional strategies that could improve collaborative learning among first-year university students, explaining the strengths and limitations of each strategy.”*

A typical AI response included suggestions such as peer-led problem-solving workshops, gamified collaborative challenges, rotating discussion leadership, project-based learning teams, and digital collaborative portfolios. Rather than accepting these responses uncritically, students were instructed to evaluate each suggestion, identify weaknesses, modify the proposed strategies where appropriate, and justify their final selections. The instructor facilitated reflective discussions by asking participants to compare AI-generated suggestions with their own ideas and explain the reasoning behind revisions. This process emphasized that AI functioned as a cognitive support tool rather than a source of authoritative answers.

Implementation fidelity was monitored through two mechanisms: (1) the instructor’s session-by-session log, which recorded attendance, the AI tool used, the activity completed, and any deviation from the planned protocol; and (2) a review of students’ independent interaction logs against the minimum threshold of two prompting cycles per week. No sessions required substantive protocol deviation. These fidelity records are treated as supplementary process documentation for this exploratory pilot rather than as a formal fidelity index; future, larger-scale replications would benefit from a standardized fidelity checklist scored by an independent observer.

The experimental group was exposed to an instructional program specifically designed to answer the study’s first research question: “What is the effectiveness of an AI-based instructional program in developing productive thinking skills among female King Faisal University students?” To address this question, the researcher reviewed a wide range of relevant literature and previous Arabic and international studies focusing on AI and productive thinking skills. Based on this foundation, the instructional program was constructed with the following components:

- Title of the instructional program: An AI-based instructional program for developing productive thinking skills among female students at King Faisal University.
- Philosophical foundation: Within this framework, the instructor primarily functioned as a facilitator, guiding students in formulating effective prompts, evaluating AI-generated outputs, and reflecting critically on their learning outcomes. Learning tasks included AI-assisted brainstorming, adaptive quizzes, collaborative concept mapping, and project-based assignments. Assessment focused on formative evaluation through continuous feedback, reflective tasks, and performance on the Productive Thinking Skills Questionnaire rather than summative grading.
- The program’s structure is based on interconnected stages that involve the careful selection of objectives, content, strategies, activities, instructional tools, and assessment methods. These are all designed to foster productive thinking skills that help students generate innovative ideas and solve problems more effectively.
- Foundational principles: The program was built upon several key pedagogical and technological principles:
- Utilization of strategies that encourage interaction, dialogue, and non-linear thinking, such as brainstorming, divergent thinking, mind maps, and problem-solving.
- Incorporation of engaging activities and evaluation techniques that promote the development of productive thinking.
- Emphasis on self-directed and continuous learning.
- Use of varied AI tools and open, rich learning environments.
- Encouragement of expressing multiple perspectives and scaffolding tasks from simple to complex.
- Continuous and holistic assessment focusing on higher-order thinking, with diverse tools and immediate feedback.
- Clearly defined roles: Instructors provide support and guidance, while students are active, independent, and engaged participants.
- Immediate training and support for students to move beyond rote application toward autonomous problem solving and decision making.
- Provision of a supportive atmosphere that respects freedom of expression, encourages curiosity, and allows time for reflection and exploration.
- General objective: The program aims to develop productive thinking skills among female students at King Faisal University.
- Specific Objectives: By the end of the program, students are expected to:
- Understand and articulate the concept of productive thinking.
- Identify AI applications relevant to productive thinking.
- Show interest and engagement in productive thinking practices.
- Use AI tools (e.g., ChatGPT, Notion AI) to generate new ideas and solve academic problems.
- Create mind maps illustrating productive thinking processes.
- Differentiate and apply various creative thinking skills, including fluency, originality, flexibility, interpretation, and inference.
- Analyze AI-generated content in terms of fluency, originality, and logical interpretation.
- Evaluate and compare AI- and human-generated outputs.
- Design innovative projects using AI tools.
- Demonstrate flexibility in adapting their thinking to evolving AI outputs.
- Apply reasoning and inference using AI-assisted analysis.
- Present AI-supported solutions to real-world problems.
- Summarize and reflect on the types and stages of productive thinking.
- Assess their own development in terms of productive thinking skills.
- Demonstrate enthusiasm for applying these skills in new contexts that utilize AI technologies.
- Instructional content: The program consisted of a series of structured sessions, each targeting a specific productive thinking skill:

Session 1: Introduction and orientation

Session 2: Developing fluency

Session 3: Developing originality

Session 4: Developing flexibility

Session 5: Developing interpretation

Session 6: Developing inference

Each instructional session was explicitly aligned with one or more dimensions of productive thinking. For example, the fluency session emphasized generating multiple responses to AI-generated problem scenarios, while the originality session focused on producing uncommon solutions evaluated against peer and AI-generated alternatives. Flexibility was addressed through tasks requiring students to reformulate solutions based on adaptive AI feedback. Interpretation and inference sessions emphasized meaning-making, pattern recognition, and drawing logical conclusions from AI-supported datasets and texts. Expansion and imagination were integrated through project-based tasks that required students to elaborate on initial ideas into feasible applications and envision alternative future scenarios. This structured alignment ensured systematic coverage of all seven productive thinking dimensions throughout the intervention.

### 3.5 Data analysis

The statistical analyses were conducted using the R programming language (version 4.3.2) and its relevant statistical packages. Descriptive statistics, including means and standard deviations, were computed to summarize participants’ responses across the study variables.

Although preliminary descriptive statistics suggested no substantial departures from symmetry, the very small sample size (*n* = 9) precluded reliable assessment of the normality assumption required for parametric procedures. Consequently, the Wilcoxon signed-rank test was selected because it provides a robust non-parametric alternative for paired observations without requiring the assumption of normally distributed difference scores. In addition to significance testing, effect sizes were calculated to determine the magnitude of the intervention’s impact. All analyses adhered to appropriate statistical assumptions and best practices for non-parametric data analysis.

The R packages used in the analysis included “psych” for computing reliability, “stats” for Wilcoxon tests, and “rstatix” for calculating effect sizes. The significance level was set at *p* < 0.05, and all statistical decisions were made based on two-tailed tests.

Effect sizes were calculated as r = Z/√N, following Rosenthal’s (1994) formula for rank-based tests, and interpreted using Cohen’s (1988) benchmarks (small ≈ 0.10, medium ≈ 0.30, large ≈ 0.50). Ninety-five percent confidence intervals for each dimension’s median pre–post difference (reported in Table 2) were computed using the exact distribution method implemented in the R stats and rstatix packages, which is appropriate for small-sample analyses and does not rely on large-sample asymptotic approximations.

## 4 Results

### 4.1 Descriptive statistics

Descriptive statistical analyses were conducted to summarize participants’ performance on the productive thinking skills scale across the pre-test and post-test administrations. The analyses included measures of central tendency (means), dispersion (standard deviations), and distributional characteristics (skewness and kurtosis) for each dimension and for the total productive thinking score. The skewness and kurtosis values across all dimensions fell within acceptable ranges, indicating that the data were approximately normally distributed and suitable for subsequent inferential analyses.

The results presented in Table 3 indicate a clear and consistent improvement in students’ productive thinking skills from the pre-test to the post-test across all measured dimensions. Pre-test mean scores reflect relatively moderate levels of productive thinking. In contrast, post-test means show substantial increases in originality, fluency, flexibility, inference, interpretation, expansion, and imagination, as well as in the overall productivity score. This upward shift suggests that students demonstrated enhanced capacity to generate ideas, adapt thinking strategies, draw inferences, interpret information, extend knowledge, and engage in imaginative reasoning following the intervention. The relatively small standard deviations across both testing phases indicate limited variability among participants, reflecting a homogeneous response pattern. Moreover, skewness and kurtosis values fall within acceptable ranges, suggesting that the score distributions approximate normality and do not exhibit problematic departures from symmetry or peakedness. Collectively, these descriptive findings indicate a marked improvement in productive thinking performance following the intervention, providing a solid basis for subsequent inferential statistical analysis.

**TABLE 3:** Descriptive statistics of productive thinking skills in the pre- and post-test.

Dimension	*N*	Mean	Std. deviation	Skewness	Kurtosis
Originality pre	9	5.89	0.601	−0.018	1.126
Fluency pre	9	8.22	0.667	−0.254	−0.04
Flexibility pre	9	3.67	0.5	−0.857	−1.714
Inference pre	9	8.11	0.601	0.018	1.126
Interpretation pre	9	3.33	0.5	0.857	−1.714
Expansion pre	9	6	0.866	0	−1.714
Imagination pre	9	4.22	0.833	−0.501	−1.275
Productivity pre	9	39.444	2.068	0.755	−0.807
Originality post	9	11.22	1.093	0.188	−1.232
Fluency post	9	14.56	1.424	−0.645	−0.543
Flexibility post	9	7.11	0.782	−0.216	−1.041
Inference post	9	14.44	1.944	−0.68	−0.234
Interpretation post	9	7.33	1.118	−0.153	−1.486
Expansion post	9	10.33	1.803	1.006	1.126
Imagination post	9	6.78	1.093	−0.188	−1.232
Productivity post	9	71.78	2.991	−0.842	0.203

### 4.2 Main hypothesis

The working hypothesis was that there would be no statistically significant difference (α ≥ 0.05) between the mean scores of the experimental group students on the pre-test and the post-test of the productive thinking scale.

**FIGURE 2:**
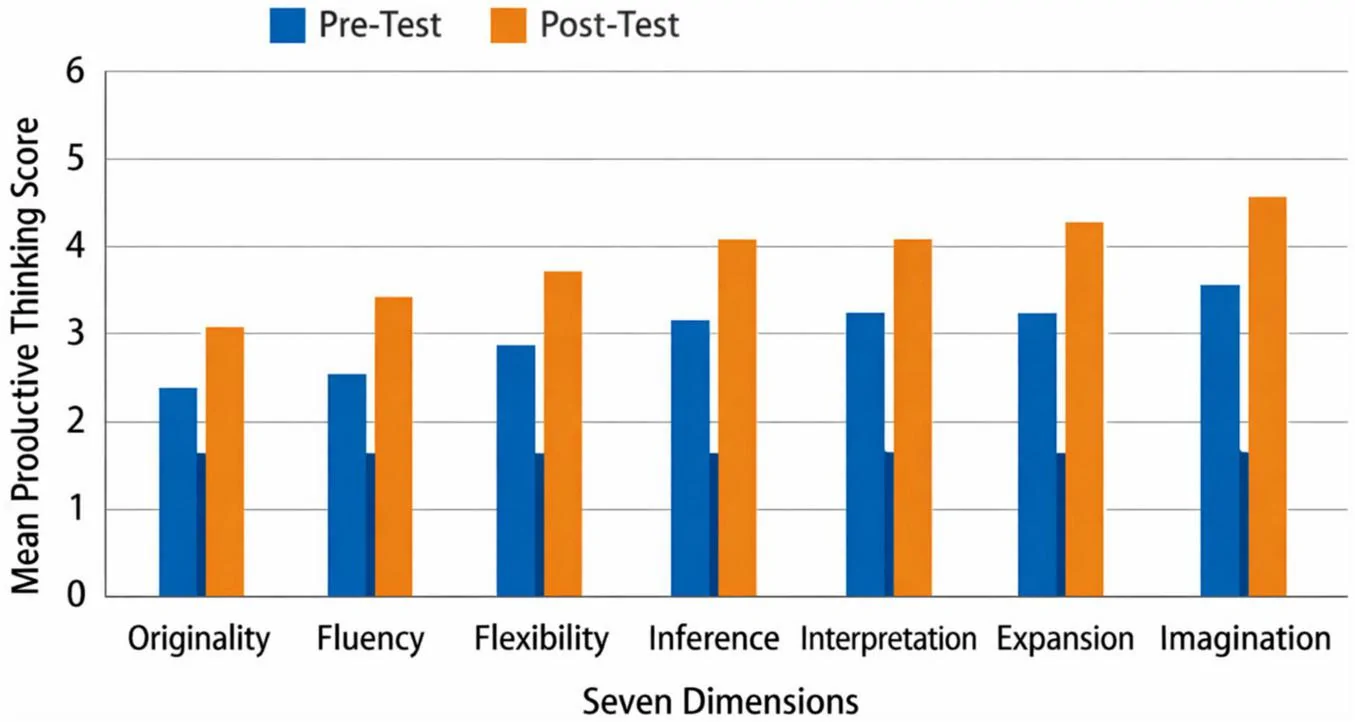
Comparison of pre-test and post-test mean scores on the productive thinking dimensions for the experimental group (*n* = 9).

To examine this hypothesis, the researcher used the Wilcoxon signed-rank test for paired samples, given the small sample size and non-parametric nature of the data. The results showed statistically significant differences between pre-test and post-test scores across all dimensions of productive thinking. Beyond statistical significance, the magnitude of the observed effect sizes indicates educationally meaningful within-group improvements, suggesting that participation in the AI-based instructional program was associated with enhanced productive thinking performance within this exploratory pilot context. The results of this analysis are presented in Table 4, and Figure 2 illustrates the differences between the pre- and post-test scores. Beyond statistical significance, the magnitude of these effects indicates meaningful educational improvement in students’ ability to generate, evaluate, and expand ideas in academic contexts.

**TABLE 4:** Wilcoxon signed-rank test results for participants’ (*n* = 9) productive thinking.

Dimension	Mean (pre)	Mean (post)	Z-value	*P*-value	Effect size (r)	95% CI (difference)
Originality	5.89	11.22	2.620	0.009	0.873	[4.15, 6.51]
Fluency	8.22	14.56	2.634	0.008	0.878	[5.02, 7.66]
Flexibility	3.67	7.11	2.639	0.008	0.880	[2.78, 4.10]
Inference	8.11	14.44	2.613	0.009	0.871	[5.11, 7.55]
Interpretation	3.33	7.33	2.620	0.009	0.873	[3.12, 4.88]
Expansion	6.00	10.33	2.618	0.009	0.873	[3.20, 5.46]
Imagination	4.22	6.78	2.460	0.014	0.820	[1.88, 3.24]
Total score	39.44	71.78	2.616	0.009	0.872	[28.4, 36.2]

Effect sizes are reported as *r* values derived from the Wilcoxon signed-rank test. According to Cohen (1988), *r* values of 0.10, 0.30, and 0.50 represent small, medium, and large effects, respectively. All observed effect sizes in this study exceed 0.80 and are therefore interpreted as large, indicating substantial educational relevance despite the exploratory nature of the study.

The results showed statistically significant differences between pre-test and post-test scores across all dimensions of productive thinking. Beyond statistical significance, the magnitude of the observed effect sizes indicates educationally meaningful within-group improvements. The results of this analysis are presented in Table 4. To facilitate visual comparison of pre-test and post-test performance across the seven productive thinking dimensions, the mean scores are illustrated in Figure 2.

### 4.3 Comprehensive analysis of productive thinking dimensions

The presented data reveal statistically significant within-group enhancements across all productive thinking dimensions, with substantial effect sizes that provide sufficient evidence to reject the null hypothesis within the constraints of this exploratory pilot study. From an educational perspective, these findings suggest that the observed improvements are not only statistically detectable but also pedagogically meaningful. The large effect sizes indicate that students demonstrated tangible gains in idea generation, cognitive flexibility, interpretive reasoning, and conceptual expansion—skills directly relevant to academic problem-solving and higher-order learning outcomes in university contexts.

### 4.4 Overall productive thinking enhancement

The aggregated productive thinking score (productivity) demonstrated a remarkable increase from a pre-test mean of 39.44 to a post-test mean of 71.78, representing an 82% improvement. This substantial enhancement is statistically significant (Z = 2.616, *p* = 0.009), with a large effect size (d = 0.872). This comprehensive improvement indicates that the intervention has profoundly influenced participants’ overall productive thinking capabilities.

### 4.5 Analysis of individual dimensions

Originality exhibited a substantial increase from 5.89 to 11.22, nearly doubling the participants’ capacity to generate novel and unique ideas. The statistical significance (Z = 2.620, *p* = 0.009) and large effect size (0.873) underscore the efficacy of the intervention in fostering creative originality.

Fluency, representing the ability to generate numerous ideas, demonstrated the highest absolute increase among all dimensions, from 8.22 to 14.56. The statistical parameters (Z = 2.634, *p* = 0.008, effect size = 0.878) confirm that participants significantly enhanced their capacity to produce abundant responses to stimuli.

Flexibility, concerning the ability to shift perspective and approach problems from multiple angles, showed a 93.7% improvement from 3.67 to 7.11. This dimension exhibited the highest effect size (0.880) of all variables, indicating that the intervention was particularly effective at enhancing cognitive adaptability.

Inference capability improved markedly from 8.11 to 14.44, indicating an enhanced ability to conclude from available information. The statistical significance (Z = 2.613, *p* = 0.009) and large effect size (0.871) affirm the intervention’s impact on logical reasoning processes.

Interpretation skills, reflecting the ability to extract meaning from information, demonstrated one of the most pronounced relative improvements, increasing from 3.33 to 7.33 (a 120% increase). The robust statistical indicators (Z = 2.620, *p* = 0.009, effect size = 0.873) validate the intervention’s effectiveness in enhancing analytical capabilities.

Expansion abilities, associated with extending and elaborating upon ideas, increased from 6.00 to 10.33. The statistical parameters (Z = 2.618, *p* = 0.009, effect size = 0.873) confirm a significant enhancement in participants’ ability to develop and enrich conceptual frameworks.

Imagination, while showing the most modest relative improvement from 4.22 to 6.78, still demonstrated a statistically significant enhancement (Z = 2.460, *p* = 0.014) with a large effect size (0.820). This suggests that, while imaginative thinking improved substantially, it may represent a more challenging cognitive domain to develop compared to other dimensions.

### 4.6 Theoretical and practical implications

The uniformly large effect sizes across all productive thinking dimensions (ranging from 0.820 to 0.880) provide compelling evidence of the intervention’s comprehensive impact on cognitive processes, thereby rejecting the study’s null hypothesis. The consistency of statistical significance (all *p*-values < 0.015) reinforces the robustness of these findings.

The hierarchical analysis reveals that flexibility and fluency exhibited the most substantial effect sizes (0.880 and 0.878, respectively), suggesting that the intervention particularly enhanced participants’ ability to generate numerous ideas from diverse perspectives. In contrast, while still exhibiting a significant effect, imagination demonstrated a comparatively lower effect size (0.820), indicating that this dimension may require additional targeted strategies for optimal development.

In conclusion, the intervention demonstrated remarkable efficacy in enhancing all dimensions of productive thinking, with particularly pronounced effects on cognitive flexibility and fluency. These findings suggest that the implemented approach effectively stimulates multiple cognitive pathways simultaneously, resulting in the comprehensive enhancement of productive thinking capabilities. The consistently large effect sizes across all dimensions validate the intervention’s robust impact on mental development, providing strong empirical support for its implementation in educational and developmental contexts.

Based on the previous results, the null hypothesis was rejected, indicating a statistically significant difference (α < 0.05) between the mean scores of the experimental group students on the pre-test and post-test of the productive thinking scale.

**FIGURE 3:**
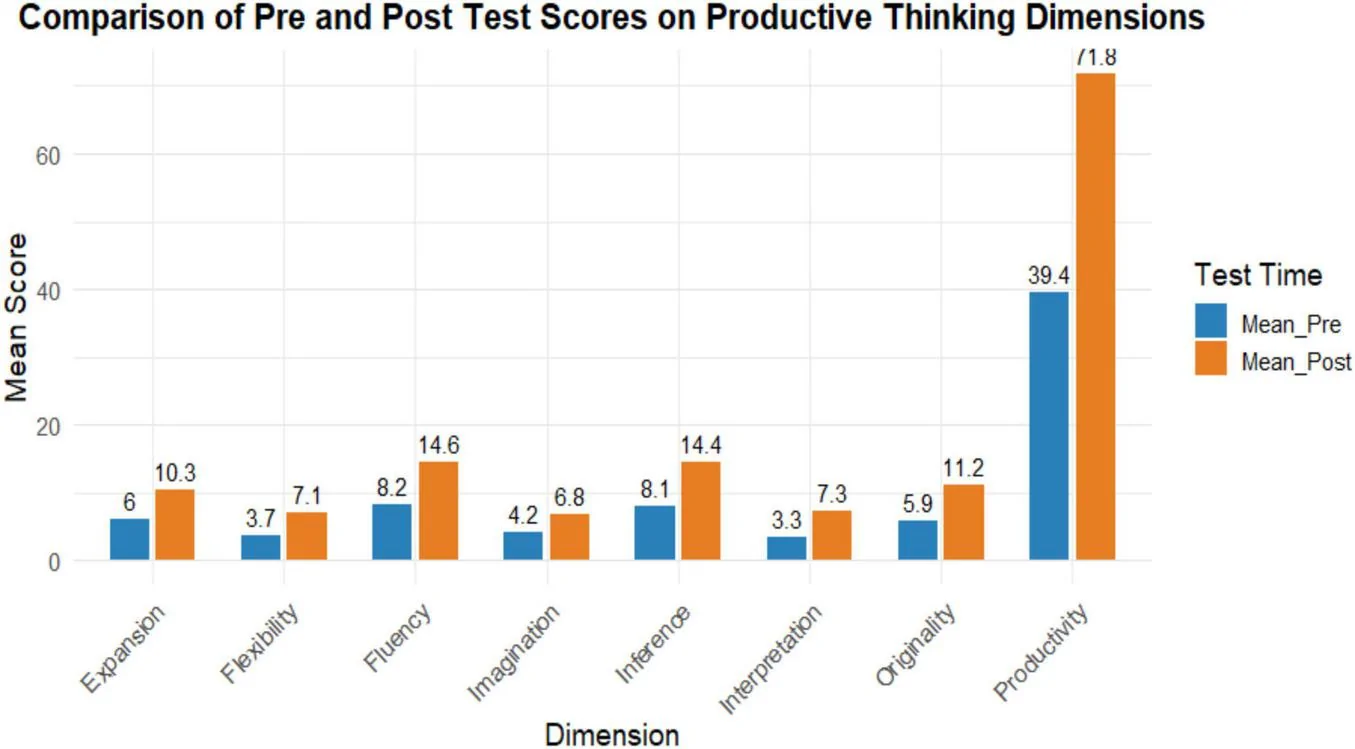
Comparison of pre-test and post-test mean scores on the productive thinking dimensions for the experimental group (*n = 9*).

The bar chart in Figure 3 provides a clear visual representation of the impact of the AI-based instructional program on the development of productive thinking skills across its seven dimensions. It reveals a consistent increase in post-test scores compared to pre-test scores among the participants, highlighting the program’s effectiveness. The total productivity score demonstrates the most significant improvement, increasing from 39.4 to 71.8, indicating a substantial overall enhancement in productive thinking abilities. Similarly, notable gains were observed in the dimensions of fluency (from 8.2 to 14.6), inference (from 6.8 to 14.4), and originality (from 5.9 to 11.2), reflecting significant improvements in the students’ ability to generate ideas, draw logical conclusions, and produce innovative responses. Dimensions such as flexibility (3.7–7.1), interpretation (3.3–7.3), and expansion (6.0–10.3) also showed evident progress, suggesting increased cognitive adaptability, interpretive understanding, and expanding ideas. Although the improvement in imagination (from 4.2 to 6.8) was relatively moderate, it nonetheless reflects growth in creative thinking. Overall, the upward trend in post-test scores across all dimensions strongly supports the conclusion that the AI-based instructional program effectively enhanced students’ productive thinking in a comprehensive and multidimensional manner.

## 5 Discussion

This exploratory pilot study examined the potential of an artificial intelligence–based instructional program to support the development of productive thinking skills among female university students. The findings indicate consistent improvements across all seven dimensions of productive thinking following participation in the AI-enhanced instructional intervention. Given the study’s small sample size and single-group design, these findings should be interpreted as associative and exploratory rather than causal, offering preliminary insights into the pedagogical potential of AI-supported learning environments rather than definitive evidence of effectiveness.

From a theoretical perspective, the observed pattern of improvement aligns with Hurson’s (2007) conceptualization of productive thinking as an integrative process that synthesizes creative and critical thinking to generate practical outcomes. The instructional design of the intervention—emphasizing idea generation, evaluation, refinement, and reflection—mirrors this conceptual integration by engaging learners in iterative cycles of divergent and convergent thinking. AI-supported activities such as guided brainstorming, adaptive feedback, and reflective comparison between human- and AI-generated outputs appear to have created conditions conducive to activating these interconnected cognitive processes.

The enhancement observed across individual productive thinking dimensions is consistent with prior research suggesting that higher-order thinking skills can be deliberately developed through structured pedagogical interventions. Improvements in fluency and flexibility align with Guilford’s (1967) foundational work on divergent thinking and with more recent studies emphasizing the responsiveness of these dimensions to instructional design that encourages multiple perspectives and adaptive problem-solving (Runco and Jaeger, 2012; Tong et al., 2023). The AI-supported tasks implemented in this study required students to generate multiple responses, revise ideas based on feedback, and approach problems from alternative viewpoints, which may explain the pronounced gains in these domains.

Similarly, the development of originality reflects existing evidence that exposure to non-traditional prompts and idea spaces—such as those generated through AI systems—can expand learners’ creative repertoires (Jordan and Mitchell, 2015; Oxman, 2006). Rather than replacing human creativity, the AI tools functioned as cognitive stimuli that challenged students to evaluate, adapt, and expand upon the generated ideas. This finding supports emerging perspectives that conceptualize AI as a cognitive partner capable of amplifying, rather than diminishing, creative thinking processes (Chiu et al., 2024). This interpretation is further supported by recent evidence from Nasr et al. (2025), who demonstrated that generative AI contributes more effectively to higher-order thinking when learners critically evaluate and refine AI-generated outputs rather than passively accepting them. Their distinction between AI-directed learning and AI-supported collaboration closely mirrors the instructional philosophy adopted in the present study, where students actively interrogated, revised, and extended AI-generated responses through structured instructor guidance. Collectively, these findings reinforce the argument that meaningful educational benefits arise not merely from access to AI technologies but from carefully designed pedagogical interactions that promote reflection, evaluation, and metacognitive regulation.

### 5.1 Theoretical interpretations of cognitive mechanisms

Beyond documenting that gains occurred, it is worth making explicit why AI-mediated interaction may plausibly produce them. Within a dual-pathway view of creative cognition, fluency- and flexibility-oriented gains are consistent with an associative, bottom-up route: rapid exposure to a wide field of AI-generated alternatives may lower the effort required to move between categories of ideas, functioning analogously to brainstorming facilitation. Gains in originality, by contrast, sit closer to a controlled, top-down route, in which students must deliberately filter, critique, and diverge from AI suggestions rather than default to them — consistent with the instructional emphasis, noted above, on treating AI outputs as a starting point for evaluation rather than a final answer.

For interpretation and inference, the mechanism is better understood as metacognitive rather than purely associative: each AI-supported activity required students to evaluate the plausibility, coherence, and limitations of a machine-generated response before acting on it, a repeated practice of externalized critical evaluation that plausibly supports the kind of reflective monitoring these two dimensions require. This reading is consistent with the study’s positioning of AI as “supported” rather than “directed” technology (Ouyang and Jiao, 2021) and offers a candidate explanation, rather than a confirmed causal account, for the pattern of gains observed across dimensions.

The observed improvements in interpretation and inference are particularly noteworthy, as these dimensions are often associated with critical thinking and deep cognitive engagement. Prior research emphasizes that the ability to extract meaning, recognize patterns, and draw reasoned conclusions is foundational to productive problem-solving (Li et al., 2022). In the present study, AI-supported analysis tasks and structured reflection appear to have facilitated these processes by encouraging students to move beyond surface-level responses toward deeper conceptual understanding. This finding resonates with constructivist perspectives that emphasize meaning-making through interaction and reflection within learning environments (Song and Cai, 2024).

Expansion and imagination, which reflect learners’ capacity to elaborate ideas and envision alternative possibilities, also showed positive development. These dimensions are closely associated with creative elaboration and future-oriented thinking, both of which are central to innovation in educational and professional contexts (Organisation for Economic Co-operation and Development [OECD], 2022). While imagination exhibited relatively minor gains compared to other dimensions, this pattern is consistent with prior research suggesting that imaginative capacity may be more resistant to short-term intervention or may require more prolonged exposure and more open-ended pedagogical conditions to develop fully (Zabelina and Robinson, 2010). Nonetheless, the observed enhancement suggests that AI-supported environments can contribute meaningfully to imaginative engagement when integrated thoughtfully into instructional design.

At a broader level, the findings align with educational frameworks that position AI within three complementary pedagogical paradigms: directed, supported, and enabling AI (Ouyang and Jiao, 2021). In this study, AI tools primarily functioned within the supported and enabling paradigms, providing guidance, feedback, and adaptive learning opportunities while maintaining students’ active role in knowledge construction. This balance appears particularly relevant for fostering productive thinking, which requires autonomy, reflection, and intellectual risk-taking rather than passive content consumption.

The results also resonate with international calls to integrate AI into education in ways that promote higher-order cognitive and lifelong learning skills (UNESCO, 2019). By situating AI use within a humanities-oriented course and a culturally specific university context, this study extends the existing literature, which has focused mainly on technical disciplines or large-scale implementations. The findings suggest that AI-supported instructional approaches can be meaningfully adapted to diverse educational settings and learner populations, including female students in non-Western higher education contexts.

Despite these promising insights, the exploratory nature of the study necessitates caution in interpreting the results. The absence of a control group and the small, homogeneous sample limit generalizability and preclude strong causal claims. Nevertheless, the consistency of improvement across all productive thinking dimensions and the alignment with established theoretical frameworks provide a compelling rationale for further investigation. Future research employing larger samples, comparative designs, and mixed-methods approaches is needed to validate and extend these findings, examine long-term sustainability, and identify the specific instructional mechanisms through which AI supports the development of productive thinking.

In sum, this discussion underscores the potential of AI-enhanced instructional environments to support multidimensional productive thinking while emphasizing the need for careful pedagogical design and empirical rigor. By framing AI as a supportive cognitive partner rather than a substitute for human reasoning, this study contributes preliminary evidence to ongoing debates on human–AI collaboration in higher education and offers a foundation for future research aimed at optimizing the educational use of intelligent technologies.

### 5.2 Limitations

Despite the promising and statistically significant findings reported in this study, several limitations must be acknowledged, which also delineate essential directions for future research. First, the absence of a control or comparison group limits the ability to attribute observed improvements in productive thinking skills exclusively to the AI-supported intervention. Although large effect sizes were observed, alternative explanations such as maturation effects, repeated testing, or concurrent academic experiences cannot be ruled out. Future research employing experimental or quasi-experimental designs with matched control groups is necessary to strengthen causal interpretations.

Second, the study was conducted with a small and relatively homogeneous sample, which restricts the generalizability of the findings. The limited sample size reduces statistical power and may not capture the variability of learning profiles present across broader university populations. Replication with larger, more diverse samples drawn from multiple institutions and academic disciplines is therefore recommended to enhance external validity and robustness.

Third, the sample composition suggests that gender- and culture-related influences on learning may have shaped participants’ engagement with AI-supported instruction. Cognitive engagement, learning preferences, and responsiveness to educational technologies can vary across gender and sociocultural contexts, yet these variables were not systematically examined in the present study. Future research should explicitly investigate gender and cultural factors as potential moderators of productive thinking development, particularly in AI-enhanced learning environments.

Fourth, the study relied exclusively on quantitative self-report measures, which, while helpful in capturing changes across productive thinking dimensions, may not fully reflect the depth, quality, or subjective nature of students’ cognitive experiences. The absence of qualitative triangulation, such as interviews, reflective journals, or classroom observations, limits interpretive richness and explanatory depth. Future studies adopting mixed-methods designs could provide more nuanced insights into how learners interact with AI tools and how these interactions influence different dimensions of productive thinking. Relatedly, because the instrument relies on self-rated Likert judgments, a construct-validity concern remains even though item-total correlation evidence from an independent pilot group (*N* = 15; *r* = 0.58\u20130.79) now supports the instrument’s internal structure: participants may still have come to perceive themselves as more productive thinkers without a fully corresponding change in demonstrated performance, since no independent, performance-based criterion measure was administered alongside the self-report scale. Such measures (e.g., rated task products, expert-scored problem-solving exercises) would help disentangle self-perceived gains from objectively demonstrated skill development and are recommended for future replications of this design.

Fifth, the study did not assess the long-term sustainability of the observed gains. Without delayed post-test or follow-up measures, it remains unclear whether improvements in productive thinking skills persist over time or diminish once the intervention concludes. Longitudinal research designs are therefore needed to examine the durability and developmental trajectory of AI-supported cognitive gains.

The intervention yielded overall positive outcomes; however, the study did not isolate or systematically document the specific instructional components responsible for enhancing particular dimensions of productive thinking. Future research should employ comparative or component-based designs to identify the active mechanisms within AI-supported instructional models. Such work would contribute to refining theory-driven instructional design and optimizing the pedagogical use of AI in higher education.

### 5.3 Future directions

Building on the current findings and in response to the identified limitations, several important directions for future research are proposed. First, future studies should employ randomized controlled and comparative research designs, incorporating both passive and active control groups engaged in alternative instructional approaches. Such comparative designs would allow researchers to determine the relative effectiveness of AI-based interventions in comparison with traditional or other innovative pedagogical methods and to isolate intervention-specific effects.

Second, future research should adopt mixed-methods approaches that integrate quantitative measures with qualitative data sources, such as semi-structured interviews, reflective journals, classroom observations, and learning artifacts. This methodological triangulation would provide deeper insight into learners’ cognitive processes, engagement patterns, and subjective experiences, thereby offering a more comprehensive understanding of how productive thinking skills develop through instructional interventions.

Third, longitudinal research designs are needed to examine the sustainability and developmental trajectory of productive thinking skills over time. Follow-up assessments conducted at multiple intervals after the intervention would help determine whether the observed gains are maintained, enhanced, or diminished, and would clarify the long-term educational impact of AI-based instructional programs.

Fourth, future studies should continue exploring individual and contextual moderators of intervention effectiveness, including baseline cognitive abilities, personality traits, motivational orientations, gender, and cultural background. Examining these factors would support the design of more personalized and context-sensitive interventions.

Fifth, advancing research on the mechanisms underlying productive thinking enhancement represents a promising direction for future exploration. Neurocognitive and neuroimaging studies could elucidate the neural correlates associated with different dimensions of productive thinking and track changes in cognitive processing resulting from instructional interventions.

Sixth, there is a need to develop and validate ecologically valid and authentic assessment tools that capture productive thinking as it occurs in real academic, professional, and everyday problem-solving contexts, rather than relying solely on decontextualized or artificial tasks.

Ultimately, future research should investigate the transfer effects of enhanced productive thinking skills on broader outcomes, including academic achievement, professional performance, and real-life decision-making. Demonstrating such transfer would strengthen the practical and societal significance of productive thinking interventions in higher education.

## 6 Conclusion

The present exploratory pilot study provides preliminary evidence that an artificial intelligence-based instructional program may support the development of productive thinking skills among female university students. Significant improvements were observed across all measured dimensions of productive thinking, suggesting that thoughtfully designed AI-supported instructional environments can foster both creative and critical thinking processes. Importantly, the intervention positioned AI as a cognitive partner that encouraged idea generation, reflection, evaluation, and refinement rather than replacing learners’ independent reasoning.

Nevertheless, the findings should be interpreted cautiously because of the small sample size, the absence of a comparison group, and reliance on self-report measures. These methodological limitations preclude strong causal conclusions but provide a valuable foundation for future investigation. Replication using larger and more diverse samples, experimental research designs, objective performance-based assessments, and longitudinal follow-up will be essential to determine the robustness, transferability, and long-term sustainability of the observed effects.

Overall, this study contributes to the growing body of research on AI-supported higher education by demonstrating the feasibility of integrating generative AI into instructional practice to promote productive thinking. As educational institutions increasingly adopt AI technologies, carefully designed pedagogical approaches that encourage active human–AI collaboration, reflective judgment, and metacognitive engagement will become increasingly important for developing the higher-order thinking skills required in twenty-first-century learning environments.

## Data Availability

The original contributions presented in this study are included in this article/supplementary material, further inquiries can be directed to the corresponding author.

## Appendix

Appendix | Sample items from the productive thinking skills questionnaire.

(The following items represent sample statements drawn directly from the data collection tool).

Originality

- The student demonstrates the ability to plan academic tasks effectively.
- The student presents functional solutions to emerging scientific problems.
- The student works on building a personal home library.

Fluency

- The student connects experiences to generate alternative solutions to problems.
- The student makes decisions appropriate to the learning situation.
- The student shares classroom concepts with family members.
- The student presents innovative ideas perceived as appropriate by peers.

Flexibility

- The student uses language clearly to express personal opinions.
- The student interacts positively with the instructional content presented by the teacher.

Inference

- The student classifies ideas presented in the lesson into multiple categories.
- The student infers new meanings from the lesson beyond those presented by the teacher.
- The student draws or represents situations studied in the lesson.
- The content enables the student to reach conceptually related inferences.

Interpretation

- The student describes encountered problems with clarity and ease.
- The student links previously used alternatives to address current solutions.

Expansion

- The student invests prior knowledge to generate realistic solutions.
- The student uses modern technologies to search for information.
- The student experiments with cognitive models proposed by the instructor.

Imagination

- The student proposes imaginative or unrealistic solutions.
- The student replaces words with other appropriate alternatives creatively.
